# Trends in innovative pediatric drug development in China based on clinical trial registration data

**DOI:** 10.3389/fmed.2023.1187547

**Published:** 2023-07-06

**Authors:** Wen-Wen Wu, Xing Ji, Xin-Shuang Mou, Xin-Yue Ma, Ya-Ting Huang, Jie-Ying Zhang, Jing-Xian Zhang, Xin-Rong Xie, Ning-Ying Mao, Jing Xu

**Affiliations:** ^1^School of International Pharmaceutical Business, China Pharmaceutical University, Nanjing, Jiangsu, China; ^2^Department of Pharmacy, Children’s Hospital of Nanjing Medical University, Nanjing, Jiangsu, China; ^3^Institute of Regulatory Science of China Pharmaceutical University, Nanjing, Jiangsu, China; ^4^National Medical Products Administration Key Laboratory for Drug Regulatory Innovation and Evaluation, Nanjing, Liaoning, China

**Keywords:** pediatric population, innovative pediatric drug, clinical trial, Chinese Clinical Trials Registry and Information Transparency Platform, research and development

## Abstract

In China, the focus of drug research and development has gradually shifted from generic to innovative drugs. Using the Chinese Clinical Trials Registry and Information Transparency Platform, we retrospectively analyzed clinical trials of innovative pediatric drugs conducted in mainland China over the last decade. The goal of this work was to better understand the characteristics of and historical changes in innovative pediatric drug research and development (R&D) in China and to provide effective data support for policy makers and other stakeholders. This study included 198 innovative pediatric drug clinical trials. The data showed that, although some progress has been made in the R&D of innovative pediatric drugs in China, many factors limiting this progress still exist, such as concentrated R&D areas, inadequate pediatric participants, and unbalanced source distributions. The level of innovative pediatric drug R&D in China currently lags behind the global level and has not kept pace with anti-neoplastic drug R&D in China. To promote the innovative development of pediatric drugs in China, the Chinese government must develop an R&D supervision framework, improve the motivation and innovation capabilities of pharmaceutical companies, and optimize the source distribution between regions.

## 1. Introduction

Based on data from the 7th Chinese Population Census in 2021, the number of individuals aged 0–14 in China is 253.38 million, accounting for 17.95% of the total population (1). According to data published by the National Medical Products Administration (NMPA) in 2016, less than 2% of the more than 3,000 listed drugs in China that year were suitable for children, whereas sick children accounted for more than 20% of the total population affected by disease. Of the more than 5,000 pharmaceutical companies in China, only 30 have products covering pediatric drugs, accounting for 0.6% (2), reflecting a serious deficiency in the level of pediatric drug development in China. Due to the special nature of pediatric growth and development, the research and development (R&D) of pediatric drugs is a global technical challenge and is influenced by various factors, such as ethics, clinical trial feasibility, industrial policy, and the market; these factors make it more difficult to guarantee the availability and safety of pediatric drugs compared to adult drugs. Therefore, solving the problem of drug development for 250 million children is an urgent challenge that China has long faced. In the past decade, the Chinese government has introduced a series of policies and regulations to encourage pediatric drug development, creating a favorable environment for the rapid development of pediatric drugs in China and increasing the number of pediatric clinical trials, with an average annual growth rate of 44.7% from 2009 to 2019, as illustrated in our previous study (3).

In our previous work, we found that most domestic companies still focused their pediatric drug R&D efforts on generic drugs with more mature markets, less investment, and shorter payback periods, and paid less attention to innovative drugs with large R&D investment, high technical difficulty, and unclear short-term market returns. However, innovative drugs with independent intellectual property patterns and novel chemical structures or new therapeutic uses undoubtedly represent the majority of the industry. Every innovative drug must go through the process of clinical trials to be commercialized; thus, the clinical trial landscape is reflective of the current development of innovative pediatric drugs in China to some extent.

All of the clinical trial data on pediatric drugs in this study are sourced from the Chinese Clinical Trials Registry and Information Transparency Platform, and through in-depth mining of the data we aim to learn more about the characteristics, historical changes, and development trends of Chinese innovative pediatric drug R&D, and to explore the effect of related incentive policies. These findings provide effective data for policymakers and other stakeholders.

## 2. Materials and methods

### 2.1. Sample source

In a previous study by our research group, we preliminarily analyzed all pediatric drug clinical trials registered on the Chinese Clinical Trials Registry and Information Transparency Platform from 2009 to 2019. In accordance with the new drug registration classification method issued by the NMPA in 2020 and the originality, novelty, and provenance of the substance structure of the drugs, innovative drugs are classified into three categories: first-in-class drugs, modified new drugs, and overseas innovative drugs. The old classification method of the Reform Scheme of the Classification System for Registration of Chemical Drugs issued by the former China Food and Drug Administration in March 2016 corresponds to the above three categories one by one according to their definitions (4, 5). The first-in-class drugs in this study must present a double innovation: they should have a “global new” substance structure and offer new clinical value. Compared with other types of drugs, first-in-class drugs require extremely high R&D capacity and industrial performance. First-in-class drugs included new classification class 1 and old classification class 1.1/1.2 chemical drugs and new classification class 1 therapeutic biological products. Modified new drugs are based upon known active ingredients with changes in the dosage, prescription technology, administration route, or indication. The key to modified new drugs lies in their “superior effect,” which requires that the changed products have obvious clinical advantages, such as enhanced efficacy, reduced adverse reactions, and improved patient compliance. Modified new drugs included new classification class 2.1 to 2.4 and old classification class 1.3 to 1.5/2/4/5 chemical drugs and new classification class 2.1 to 2.4 therapeutic biological products. Overseas innovative drugs refer to drugs with overseas original research listings that are applying for listing in China (the first drug approved for listing with complete and sufficient safety and effectiveness data is the basis for listing). Overseas innovative drugs included new classification class 5.1 chemical drugs and new classification class 3.1 therapeutic biological products. The specific classifications are provided in the Supplementary Table S1. In addition, due to significant differences in the definition and defined scope of innovative drugs between Chinese/natural medicines and prophylactic biological products, chemical drugs, and therapeutic biological products, no direct comparison could be made. The development and the approval process for prophylactic biological products are also different from those for therapeutic drugs. Thus, this study did not conduct a detailed analysis of Chinese medicines, natural medicines, and prophylactic biological products.

### 2.2. Retrieval strategies and data analyses

The data source for this study was the Chinese Clinical Trials Registry and Information Transparency Platform (hereafter referred to as the platform). In 2013, the NMPA Center for Drug Evaluation established the platform based on the “Chinese Drug Clinical Trial Database” and mandated that all drugs that have received clinical trial approval and have been tested in clinical trials in China be registered and their information displayed on the platform. The contents of the platform were designed based on ICH-definitions and ICH-term. It requires all clinical trials conducted in China with clinical trial approvals from the National Medical Products Administration (including bioequivalence trials, PK trials, Phase I-IV trials, etc.) to be registered and publicly disclosed on the platform with specific registration deadlines. For clinical trials conducted before 2013 and for which new drug applications have not yet been completed, retrospective registration is required. The clinical trial data provided by the “Chinese Clinical Trials Registry and Information Transparency Platform” is comprehensive, reliable, and timely. Therefore, the pediatric innovative drugs’ clinical trial data involved in this study are all sourced from this platform.

We conducted a retrospective study of clinical trial information registered on the platform before December 31, 2021. After the preliminary retrieval, 15,522 registered clinical trials were identified. This study included all clinical trials of innovative pediatric drugs from the registered clinical trials mentioned above. Retrieval and data processing were divided into the three steps outlined below.

The first step involved screening clinical trials that involved pediatric indications and were conducted by institutions specializing in pediatric drugs. Firstly, referring to the WHO’s classification of age groups for children, the search terms for indications were set as “newborn” “infant” “child, preschool” “child” “adolescent” and “teenager” all of which are MeSH terms. By the Chinese practice of formulating indications, we added “infant and toddler” as a search term. The terms “child” and “maternal and child” were used as search terms for clinical trial institutions. Therefore, the search terms for the first step included “newborn” “infant” “infant and toddler” “child, preschool” “child” “adolescent” “teenager” and “maternal and child.” After the first selection, 1,315 clinical trials were included. We screened 401 trials with duplicate platform registration numbers, 45 trials with duplicate common titles (Duplications were due to different dimensional retrieval strategies.) and 21 trials only include adults. Two pediatricians independently reviewed the screened clinical trials, and if there was any disagreement, a third pediatrician was asked to arbitrate until a consensus was reached. This step excluded 52 non-pediatric clinical trials. To confirm that no relevant trials were missed, 10% of the trials were selected from the excluded group (*n* = 519) using random sampling. No pediatric-related clinical trials were found after a review by the two pediatricians mentioned above. A total of 796 pediatric clinical trials were included after the first screening step.

**Figure 1:**
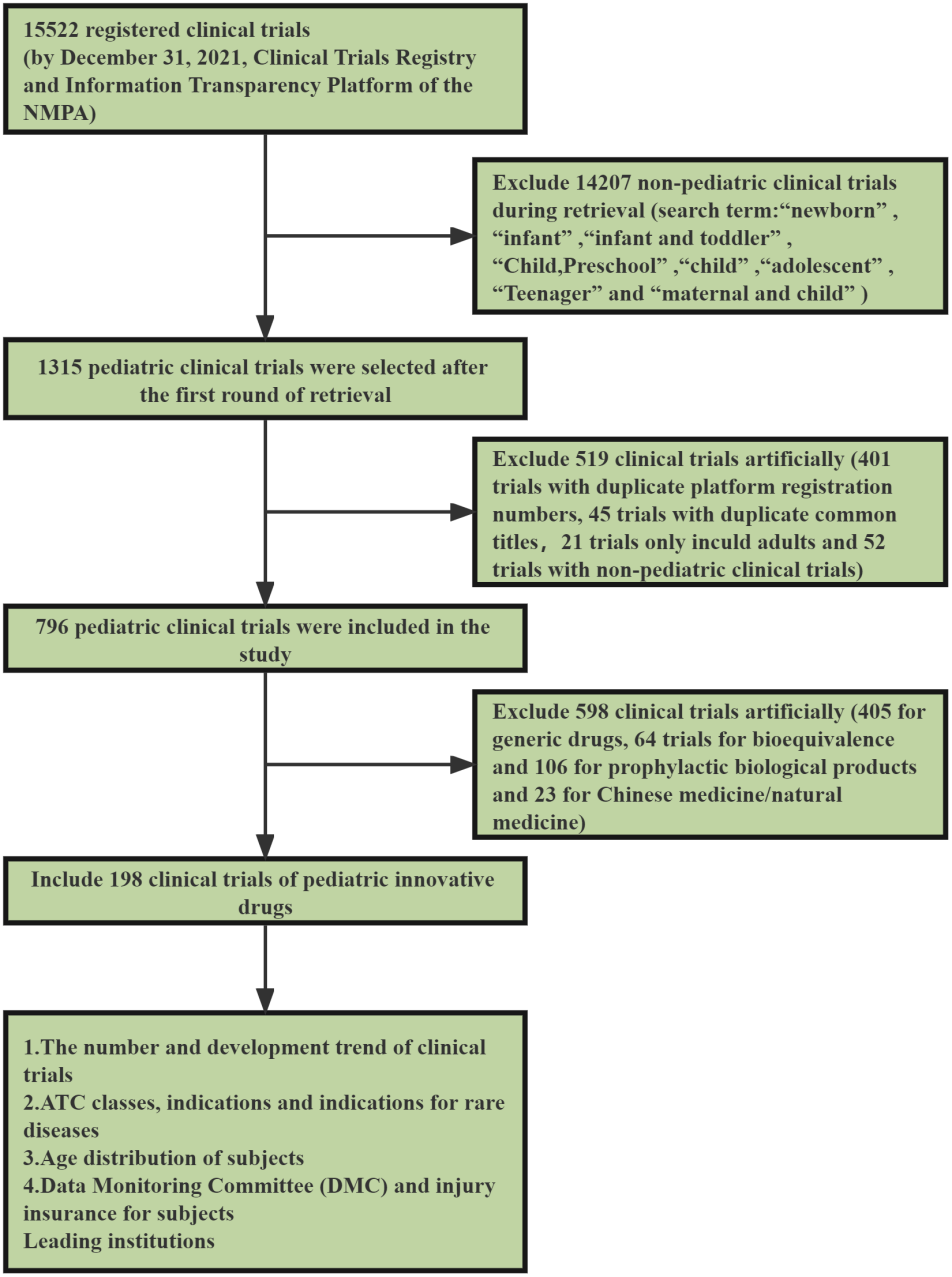
Strategy of retrieval and selection of pediatric clinical trials.

The second step involved screening the list from step one for clinical trials of innovative pediatric drugs. In accordance with the latest 2020 version of the Provisions for Drug Registration and the category of innovative drugs in the classification guidelines for drug registration, two pediatricians in the research group independently decided whether the registration classes of the drugs under clinical trials were innovative or not. This step excluded 405 clinical trials registered as generic drugs, 64 registered as bioequivalence, 106 registered as prophylactic biological products, and 23 registered as Chinese/natural medicines. In total, 598 clinical trials were excluded, leaving 198 innovative drug trials. To confirm that the drugs in these clinical trials were all innovative, 20% of the 198 innovative drug trials included were selected using a random sampling method and confirmed as innovative drug clinical trials by a third pediatrician. Ultimately, our study included 198 innovative pediatric drug clinical trials. See Figure 1 for specific screening steps.

The third step involved data analysis and statistical analysis. Microsoft Excel and SPSS were used for data processing and analyses. Descriptive analyses were performed on the number of innovative pediatric drug clinical trials, trial phases, innovative drug classes, registration types, drug anatomical therapeutic chemical (ATC) classes, indications, age distribution of children participating in the trials, orphan drugs, and host institutions. Qualitative variables were expressed as percentages. Simple regression models have been used to analyze the development trends of the items mentioned above in recent years. Annual rates of change were calculated for each indicator, and the year in which the trial belonged was determined using the date of the first ethics review.

## 3. Results

### 3.1. Basic information regarding innovative pediatric drug clinical trials

**Figure 2:**
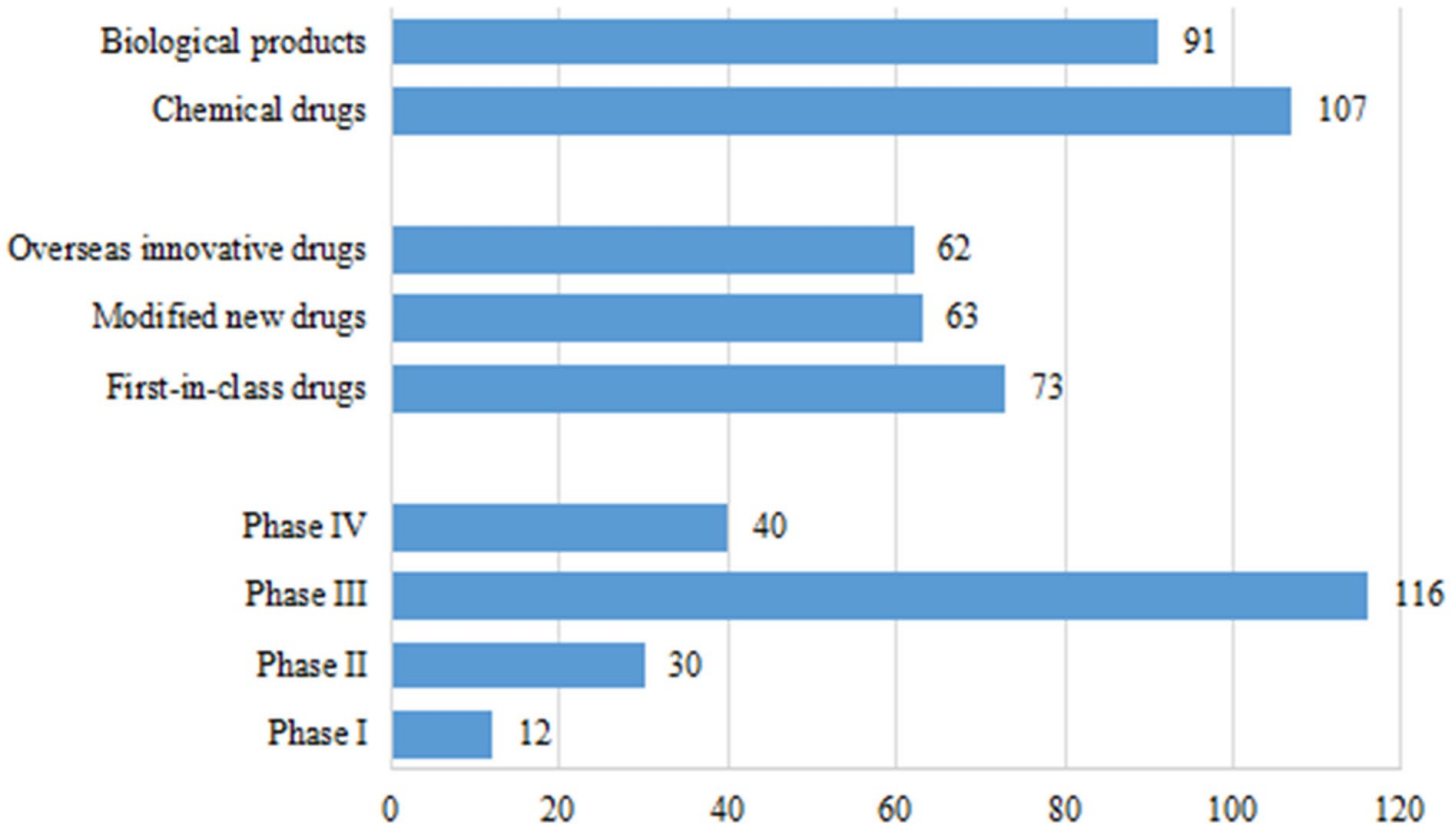
Drug types, trial phases, and registration types of 198 clinical trials for pediatric innovative drugs in China.

As of December 31, 2021, a total of 796 pediatric clinical trials had been conducted in mainland China. There were 198 innovative clinical trials for chemical drugs (107, 54.0%) and therapeutic biological products (91, 46.0%), accounting for 24.9% of all pediatric clinical trials. With regards to trial phase, the majority of trials were Phase III (116, 58.6%). There were only 12 trials in Phase I (6.1%), 30 in Phase II (15.2%), and 40 in Phase IV (20.2%). Of the drug registration types, there were 73 first-in-class (36.9%), including 31 chemical drugs and 42 therapeutic biological products; 63 modified new drugs (31.8%), including 34 chemical drugs and 29 therapeutic biological products; and 62 overseas new drugs (31.3%), including 42 chemical drugs and 20 therapeutic biological products (Figure 2).

**Figure 3:**
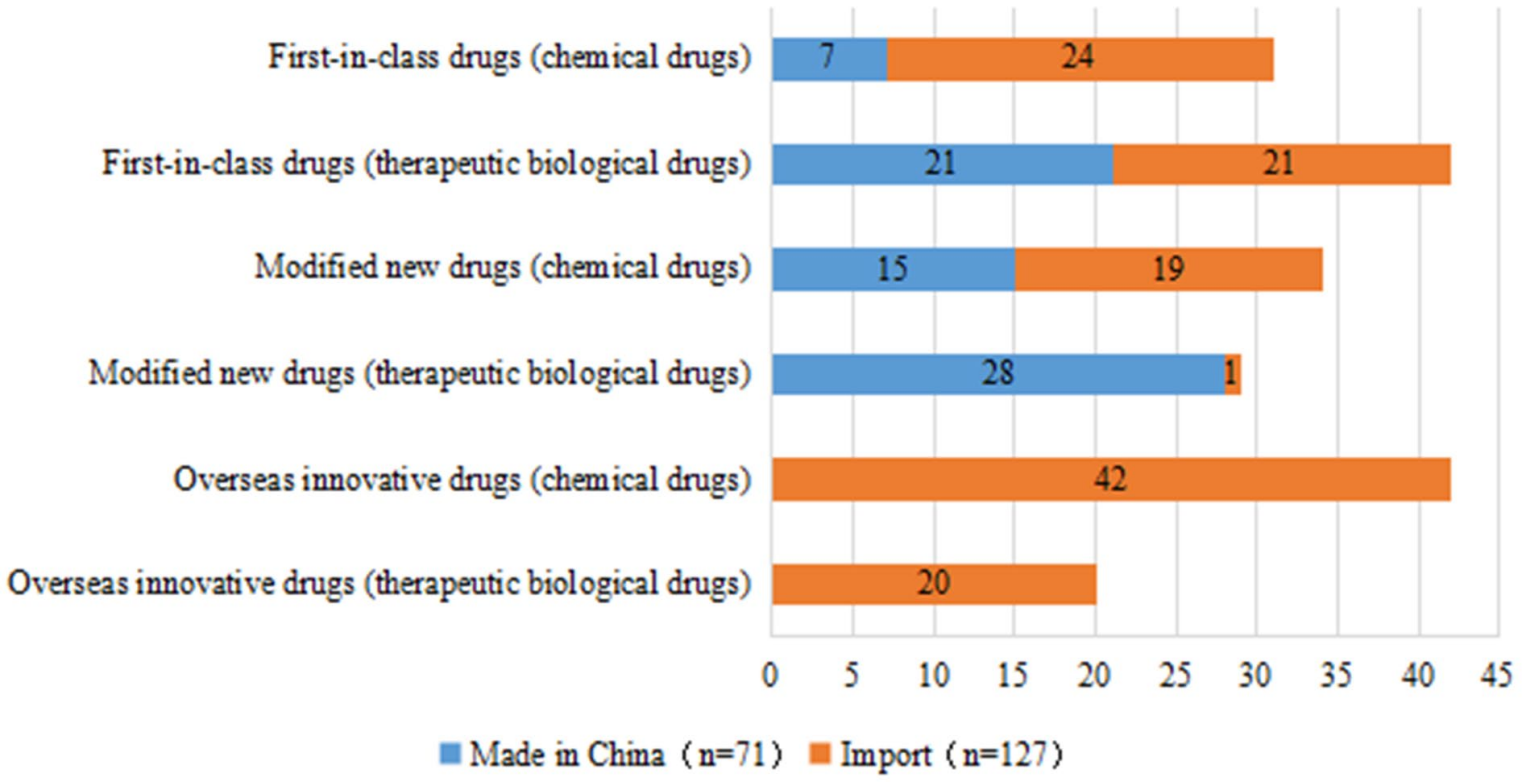
Distribution of R&D companies corresponding to clinical trials of pediatric innovative drugs by registration class.

In terms of R&D sponsors, 78 trials were conducted by domestic companies (39.4%) and 120 trials were conducted by overseas companies (60.6%). Of the 73 first-in-class drug trials, 28 were conducted by domestic companies (38.4%), most of which involved therapeutic biological products (75.0%), with only 7 trials for chemical drugs (25.0%). Forty-five of the 73 first-in-class trials were conducted by overseas companies, comprising 24 trials for chemical drugs and 21 for therapeutic biological products. The number of trials for modified new drugs initiated by domestic companies (43, 68.3%) was much higher than that by overseas companies (20, 31.7%). From a different perspective, of the 71 clinical trials initiated by domestic companies, 49 (69.0%) were for therapeutic biological products. Clinical trials initiated by overseas companies focused more heavily on chemical drugs, with only 42/127 trials (33.1%) focused on therapeutic biological products (Figure 3). If an overseas institution was involved in one clinical trial, this trial will be considered an international multi-center trial. Of the 198 clinical trials, 78 were international multi-center trials initiated by overseas companies (39.4%).

### 3.2. Trends in innovative pediatric drug development run-up time

**Figure 4:**
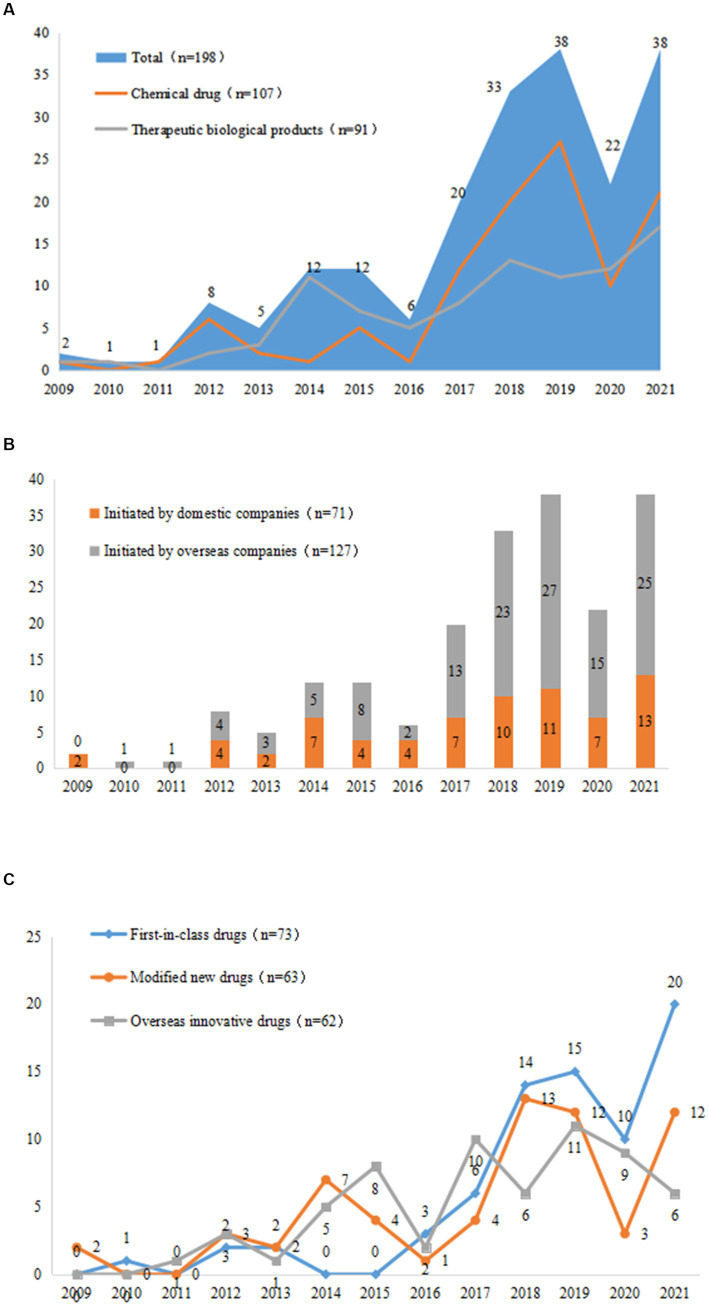
Changes in trends of drug types **(A)**, R&D companies **(B)**, and registration types **(C)** in 198 pediatric innovative drug clinical trials in China.

From 2009 to 2021, the number of clinical trials for innovative pediatric drugs gradually increased, with an average annual growth rate of 27.8%; from 2017 to 2019, three-year-on-year growth rates of 70.0, 39.4, and 13.2%, respectively, were achieved. The number of trials initiated in 2019 was 38, which was the highest in history. This number declined to 22 in 2020, which may have been related to the global COVID-19 pandemic. The number of innovative drug clinical trials initiated in 2021 has increased once again, returning to the 2019 level. The growth trends of clinical trials for chemical drugs and therapeutic biological products have been nearly identical, with growth rates of 28.9 and 27.2%, respectively, since 2009; however, since 2017, the growth rate and sheer number of chemical drug trials have been much higher than those of therapeutic biological products. The number of chemical clinical trials in 2020 significantly decreased to only 37.0% of that in 2019, while, in the same year, the number of therapeutic biological products was unaffected by COVID-19 (Figure 4A).

The average growth rate of clinical trials initiated by domestic companies was 16.9%, which was much lower than that of overseas companies (34.0%). Notably, domestic companies were less affected by the COVID-19 pandemic, as the number of clinical trials initiated domestically decreased by 36.3%, while overseas companies experienced reduction of 44.4% (Figure 4B).

In terms of drug registration classes, the annual growth rates of first-in-class drugs, modified new drugs, and overseas innovative drugs were 31.3, 16.1 and 25.1%, respectively, and the total number of drugs increased significantly after 2017. Modified new drugs were particularly affected by COVID-19: the number of these in 2020 was only 25.0% of that in 2019, and the number of domestic and foreign companies was 33.3 and 16.7%, respectively, of the previous year. Comparatively, first-in-class drugs and overseas innovative drugs were less affected than modified new drugs. In particular, the number of clinical trials of first-in-class drugs initiated by domestic companies did not decline in 2020 (Figure 4C).

### 3.3. ATC classes and indications involved in clinical trials of innovative pediatric drugs

#### 3.3.1. ATC classes

**Figure 5:**
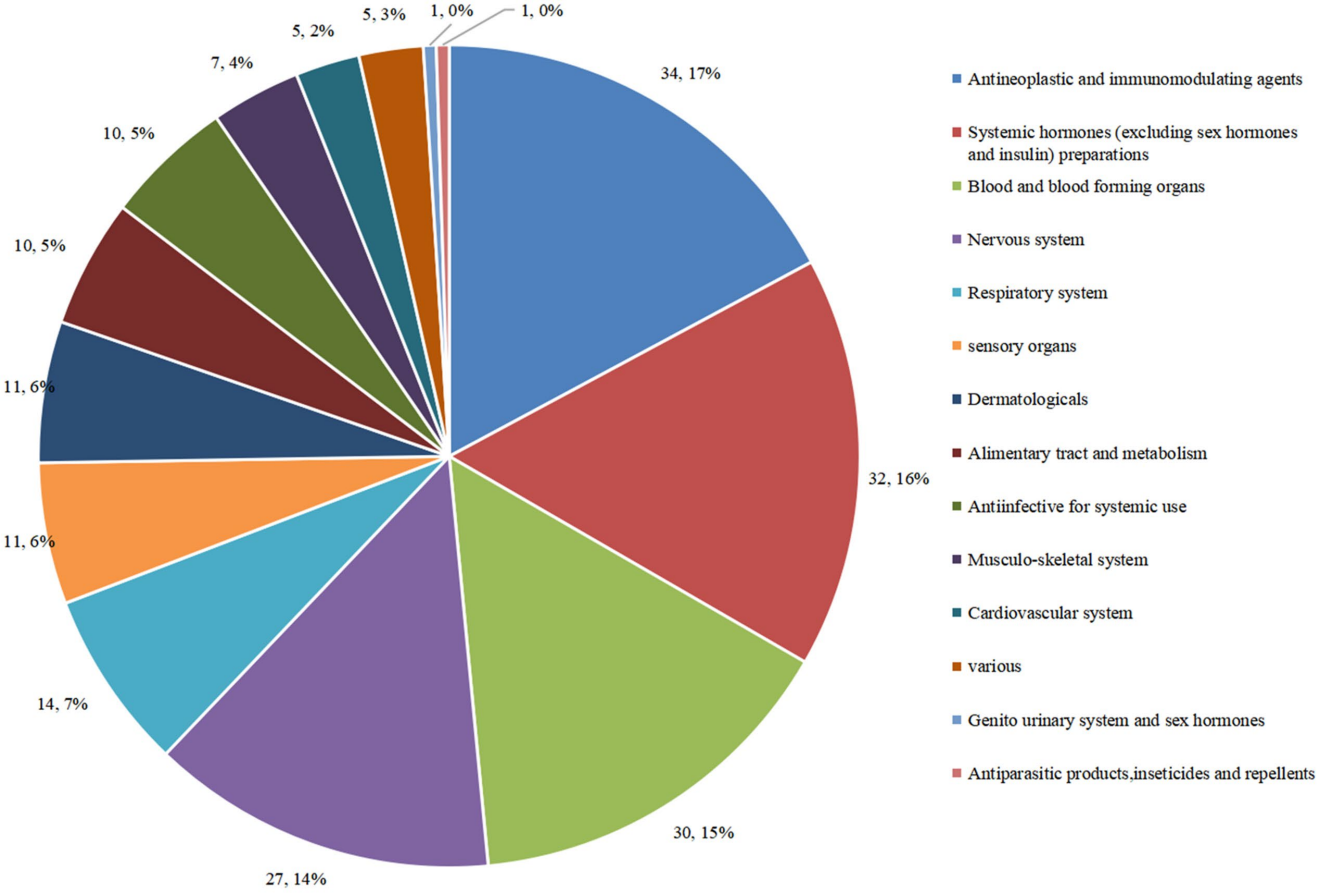
Distribution of clinical trials corresponding to different ATC classes of pediatric innovative drugs in China.

To some extent, ATC classes and indications of drugs in clinical trials may reflect a hot field of drug R&D. Here, we analyzed the ATC classification of innovative drugs in pediatric clinical trials based on the official system for drug classification used by the World Health Organization (WHO)-ATC system. The data showed that the 198 trials covered 14 ATC classes, 61 indications, and 130 drug types. Of the 198 trials, the number of trials for anti-neoplastic and immunomodulatory agents was the largest, comprising 34 trials (17.2%). The next top four classes were systemic hormones (excluding sex hormones and insulin) preparations (16.2%); blood and blood-forming organs (15.2%); nervous system (13.6%); and sensory organs (7.1%) (Figure 5).

**Figure 6:**
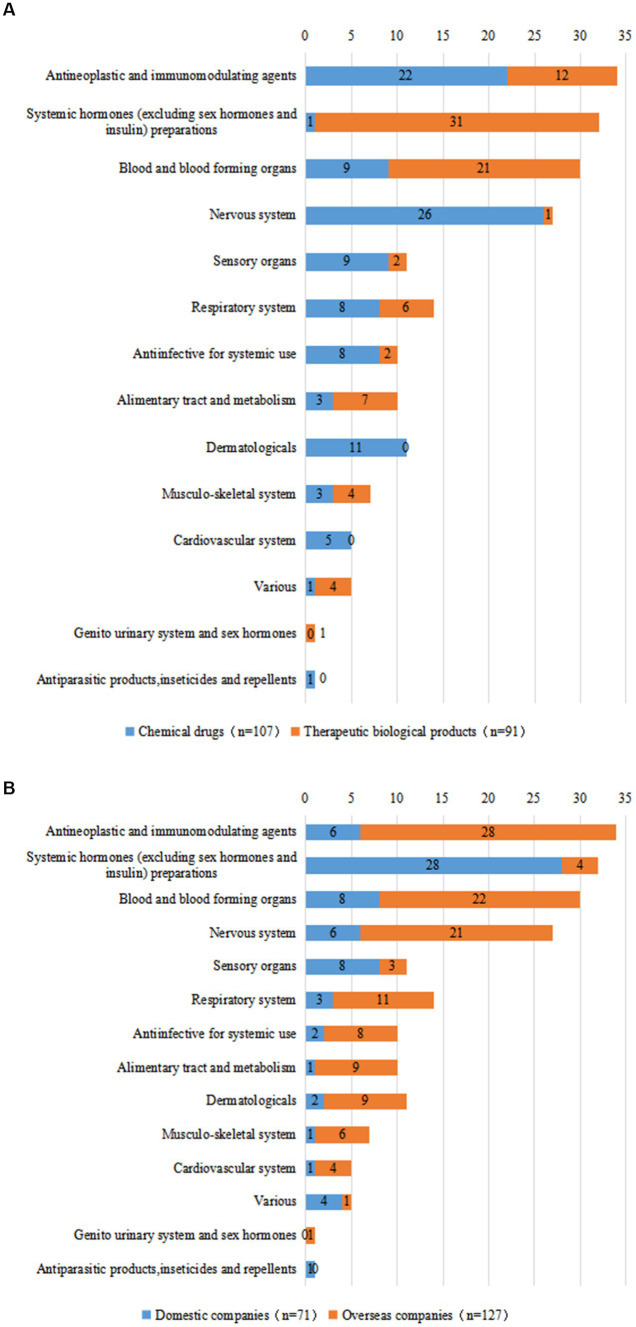
Distribution of clinical trials corresponding to ATC classes of different drug types **(A)** and R&D companies **(B)** of pediatric innovative drugs in China.

Subgroup analysis was conducted for each drug type, and the ATC classification of chemical drugs and biological products was analyzed. A total of 107 clinical trials were conducted on chemical drugs and 91 on biological products. ATC classes of chemical drugs specifically were mainly concentrated in the areas of the nervous system (24.3%), anti-neoplastic and immunomodulating agents (20.6%), and sensory organs (10.3%). Those of 91 therapeutic biological products were mainly concentrated on systemic hormones (excluding sex hormones and insulin) preparations (34.1%); blood and blood-forming organs (23.1%); and anti-neoplastic and immunomodulating agents (13.2%) (Figure 6A).

According to the subgroup analysis conducted by the sponsor for domestic or overseas enterprises, 71 clinical studies were initiated by domestic enterprises and 127 by overseas enterprises. We observed a significant difference in the research fields of domestic and overseas companies. The types of innovative drugs developed by domestic companies were mainly concentrated on systemic hormones (excluding sex hormones and insulin) preparations (39.4%); sensory organs (11.3%); and blood and blood-forming organs (11.3%). The innovative drugs developed by overseas companies were concentrated on anti-neoplastic and immunomodulating agents (22.0%), blood and blood forming organs (17.3%), and the nervous system (16.5%) (Figure 6B).

#### 3.3.2. Indications

This study comprehensively analyzed the indications for innovative drugs based on the classification rules for drug indications in *Chinese National Formulary.* The 198 clinical trials covered 61 indications, the top 5 of which were growth retardation in children (14.1%), hemophilia A (8.1%), epilepsy (7.1%), asthma (6.1%), and atopic dermatitis (4.5%). This finding was similar to those of our previous work in which we considered all pediatric clinical trials (3). In addition, there are six indications with a clinical trial count of 5–8: juvenile idiopathic arthritis (6), myopia (7), tumor (7), syncytial virus infection (5), hemophilia (not specified) (5), and pulmonary artery hypertension (5) (Supplementary Table S1).

**Figure 7:**
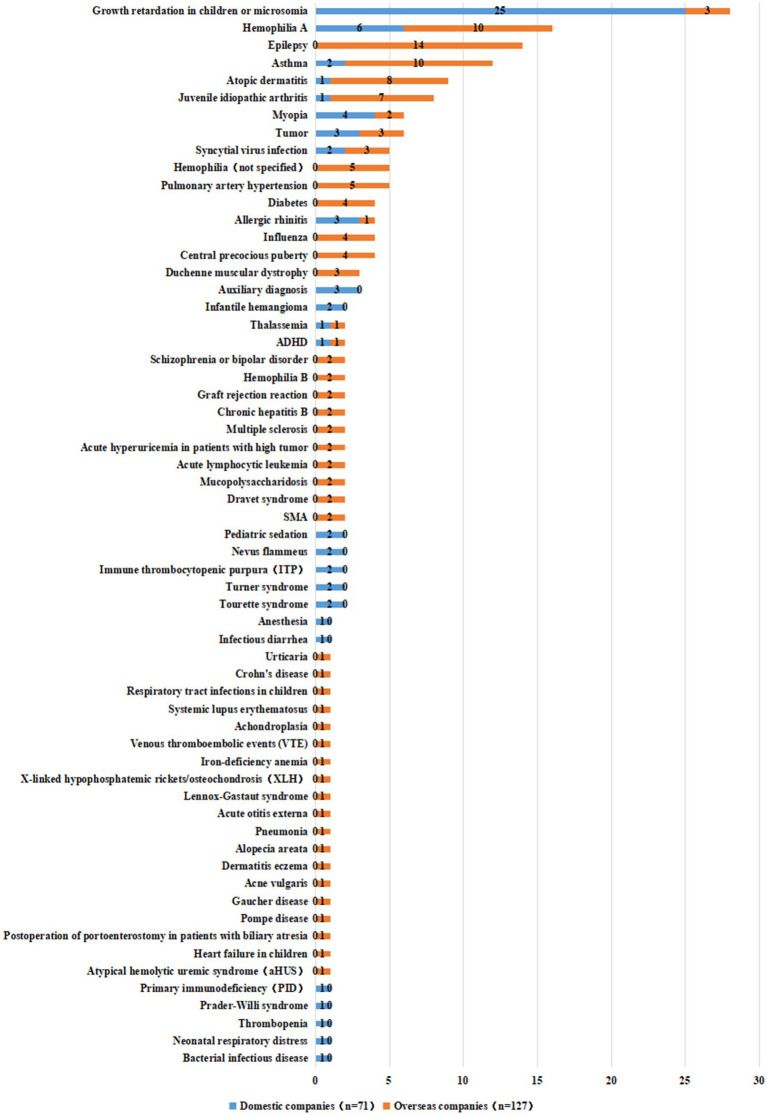
Distribution of indications of 198 pediatric innovative drug clinical trials initiated by different R&D companies in China.

We also observed differences in the indications for drugs developed by domestic and international companies. Subgroup analysis according to the sponsor for domestic enterprises or overseas enterprises revealed that innovative drugs developed by domestic companies covered 25 indications (in 71 clinical trials), of which the most common was growth retardation in children with 25 trials accounting for 35.2% of all clinical trials by domestic companies. The next most common indications were hemophilia A with only six trials, accounted for less than 8.4% of all trials. The number of clinical trials corresponding to other indications is all less than 5. Conversely, innovative drugs developed by overseas companies covered 47 indications (in 127 clinical trials), among which clinical trials were evenly distributed. The most common indication was epilepsy with 14 trials accounting for 11.0%. The next most common indications were hemophilia A and asthma, with 10 trials each. Three indications had more than 10 clinical trials and seven had more than five clinical trials (Figure 7).

#### 3.3.3. Indications for rare diseases

In accordance with the First List of Rare Diseases released in May 2018 and the List of References on Rare Diseases in China (7, 8), we screened clinical trials of innovative drugs indicated for rare diseases and identified 52 trials, accounting for 26.3% of all clinical trials, which involved 34 drug types. The largest number of trials was for hemophilia (23 trials, 44.2%): 16 trials focused on hemophilia A, two on hemophilia B, and five were not specified. The remaining 29 clinical trials of drugs for rare diseases involved indications for pulmonary hypertension (5 trials), followed by Duchenne muscular dystrophy (3 trials) (Table 1). In terms of orphan drug registration classes, the numbers of clinical trials for first-in-class drugs, modified new drugs, and overseas innovative drugs were 21, 13, and 18, respectively. In terms of R&D institutions, there were only 13 clinical trials developed and initiated by domestic companies (25.0%), covering six rare disease indications, and nearly half of those trials were for hemophilia (46.2%). There were 39 clinical trials developed and initiated by overseas companies (75.0%), covering 16 indications (76.2%) and involving a wider range of drug indications than those of domestic companies.

**Table 1:** Distribution of ATC classes, indications, and number of trials for rare diseases (52 clinical trials) in 198 pediatric innovative drug clinical trials.

ATC classes	Indication	No. of trials
Blood and blood-forming organs	Hemophilia A	16
	Hemophilia (not specified)	5
	Pulmonary arterial hypertension	2
	Hemophilia B	2
	Transfusion-dependent β-thalassemia	1
Nervous system	Dravet syndrome	2
	Tourette syndrome	2
	Rare epilepsy	1
	Lennox–Gastaut syndrome	1
Musculo-skeletal system	Duchenne muscular dystrophy	3
	SMA	2
Alimentary tract and metabolism	Mucopolysaccharidosis	2
	Gaucher disease	1
	Pompe disease	1
Systemic hormones (excluding sex hormones and insulin) preparations	Turner syndrome	2
	Prader-Willi syndrome	1
Antineoplastic and immunomodulating agents	Multiple sclerosis	2
	Crohn’s disease	1
Cardiovascular system	Pulmonary arterial hypertension	3
Others	Thalassemia	1
Respiratory system	Neonatal respiratory distress	1

### 3.4. Age distribution of children participating in clinical trials of pediatric innovative drugs

In accordance with the WHO classification of children’s age, the subjects participating in the clinical trials were classified as newborns (<28 days), infant (1–23 months), child (2–11 years), adolescent (12–17 years), and adults (18 years and above). Previous studies have shown that a primary challenge of conducting pediatric clinical trials is the recruitment of pediatric subjects. Of the 198 clinical trials of innovative pediatric medicines included in this study, 111 clinical trials included adult subjects (56.1%), while 87 trials (43.9%) were limited to adolescents and younger (Table 2).

**Table 2:** Age distribution of subjects in 198 clinical trials of pediatric innovative drugs in China.

Age groups of subjects		No. of trials	%
Subjects included adults	Adolescents, adults	40	20.2
	Adolescents, children, adults	38	19.2
	Infants, children, adolescents, adults	11	5.6
	Neonates, infants, children, adolescents, adults	22	11.1
Subjects included, no adults	Neonates	1	0.5
	Neonates, infants	3	1.5
	Neonates, infants, children	5	2.5
	Neonates, infants, children, adolescents	5	2.5
	Infants	2	1.0
	Infants, children	5	2.5
	Infants, children, adolescents	7	3.5
	Children	18	9.1
	Children, adolescents	40	20.2
	Adolescents	1	0.5
Total		198	100

### 3.5. Data monitoring committees for pediatric innovative drugs

A Data Monitoring Committee (DMC) is an organization that protects the safety of subjects, ensures trial reliability and the validity of results throughout the clinical trial process, and is particularly important for the protection of all parties involved in pediatric clinical trials. Fifty-eight of the 198 clinical trials for innovative drugs (29.3%) established a DMC. From 2012 to 2021, the overall rate of establishing a DMC for innovative pediatric drug clinical trials in China steadily increased, with an annual growth rate of 34.1%. In our previous study, from 2011 to 2019, only 9.9% of 487 pediatric clinical trials established a DMC (3). The rate of DMC establishment in pediatric clinical trials of innovative drugs is higher than that of non-innovative drugs.

**Figure 8:**
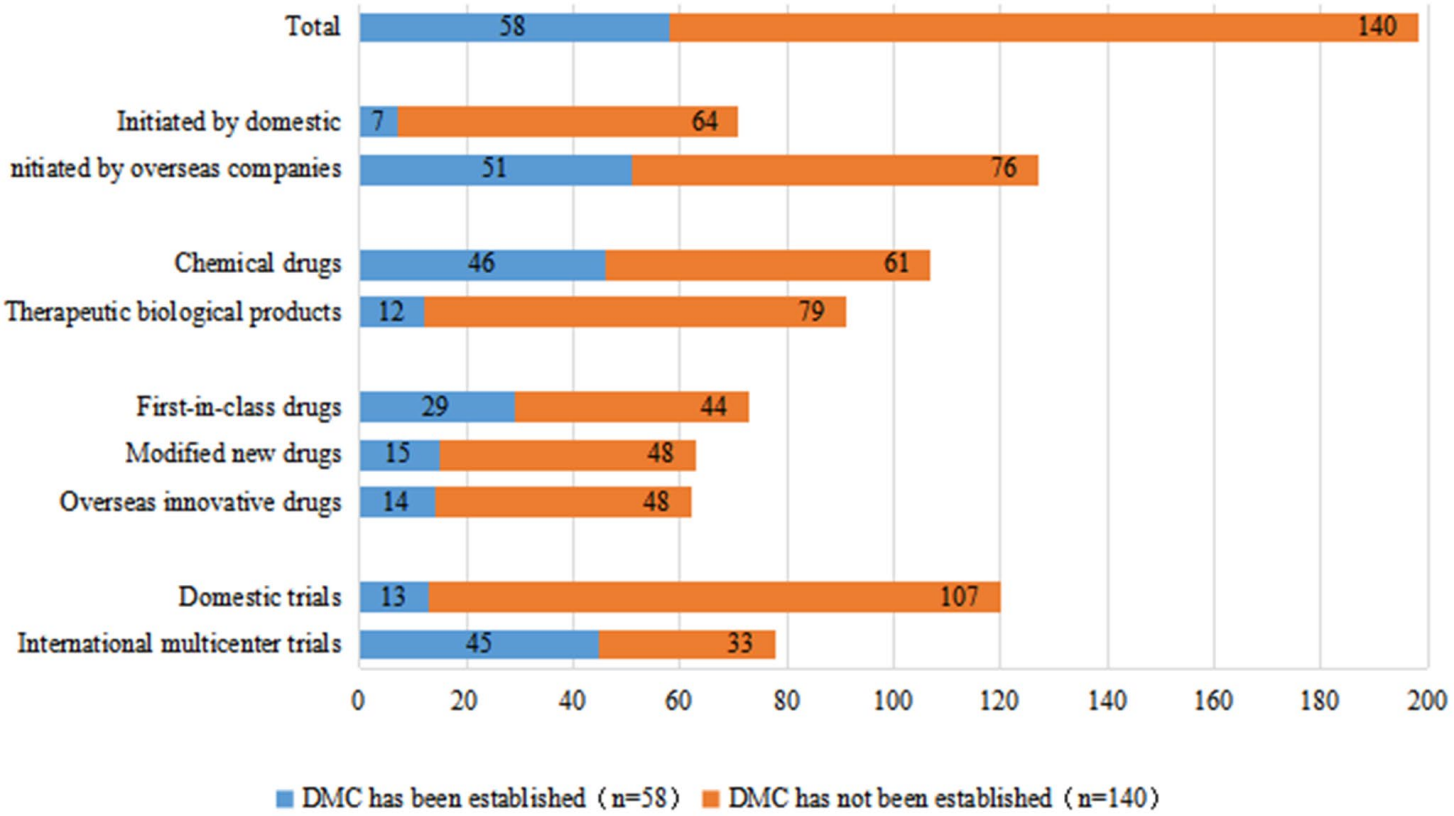
DMC establishment for 198 clinical trials of pediatric innovative drugs in China.

However, from the perspective of the sponsor, similar to all pediatric clinical trials, there is still a large gap between domestic and foreign companies in terms of DMC establishment. Only 7 of 71 clinical trials (9.8%) initiated by domestic companies established a DMC, while 51 of 127 clinical trials (40.2%) initiated by overseas companies established a DMC. Furthermore, 45 of 78 international multi-center trials (57.7%) established a DMC, but only 13 of 120 domestic multi-center trials (10.8%) established a DMC. DMC establishment rates for clinical trials of chemical drugs and therapeutic biological products were 43.0 and 13.2%, respectively. DMC establishment rates for clinical trials of first-in-class drugs (39.7%) were higher than those of modified new drugs (23.8%) and overseas innovative drugs (22.6%) (Figure 8).

### 3.6. Injury insurance for innovative pediatric drug clinical trial subjects

Insurance is an important safeguard measure for the rights and interests of clinical trial participants and an important risk-sharing mechanism for sponsors. Of the 198 clinical trials of innovative pediatric drugs, 153 trials (77.3%) had injury insurance for subjects; this was more than 30% higher than the 44.8% overall insurance rate in pediatric clinical trials reported in our previous study (3), indicating that sponsors of innovative pediatric drugs have become significantly more aware of purchasing insurance for their subjects. The rising trend in insurance coverage for innovative pediatric drug clinical trials in China was even more pronounced, with an annual growth rate from 2010 to 2021 of 38.9%.

**Figure 9:**
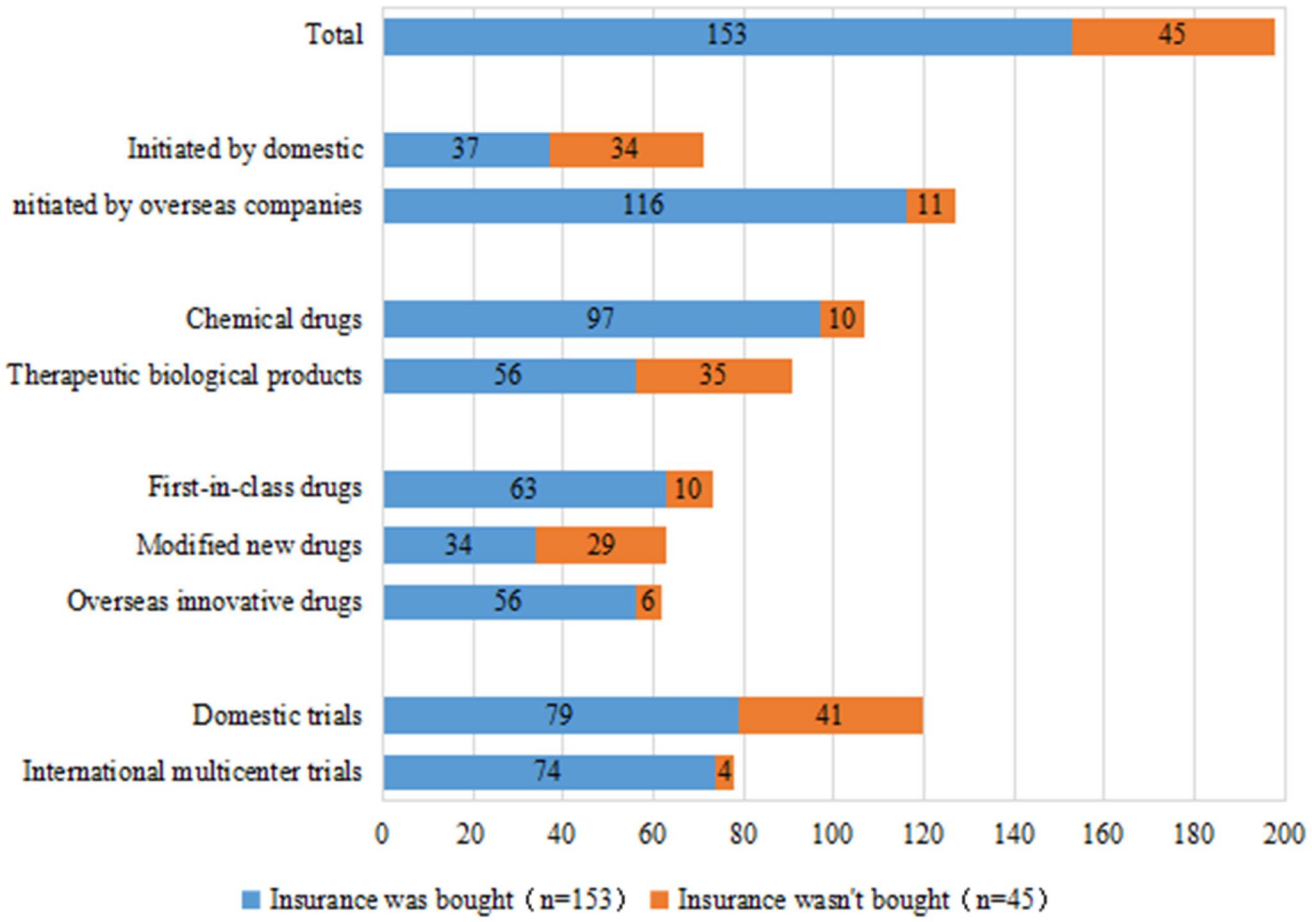
Injury insurance for subjects in 198 clinical trials of pediatric innovative drugs in China.

Similar to the rate of DMC establishment, we observed a gap in the insurance rate of clinical trials initiated by domestic versus overseas companies: 52.1% (37/71 trials) and 91.3% (116/127 trials), respectively. With regards to international multi-center trials, 94.9% (74/78 trials) had injury insurance for subjects, compared to only 65.8% (79/120 trials) of domestic trials. The insurance rates for clinical trials of chemical drugs and therapeutic biological products were 90.6% (97/107 trials) and 61.5% (56/91 trials), respectively. Clinical trials of first-in-class drugs and overseas-marketed drugs had comparable rates of insurance coverage for subjects at 86.3% (63/73 trials) and 90.3% (56/62 trials), respectively, whereas the insurance rate for subjects in clinical trials of modified new drugs was lower at only 54.0% (34/63 trials) (Figure 9).

### 3.7. Leading institutions of innovative pediatric drug clinical trials

**Figure 10:**
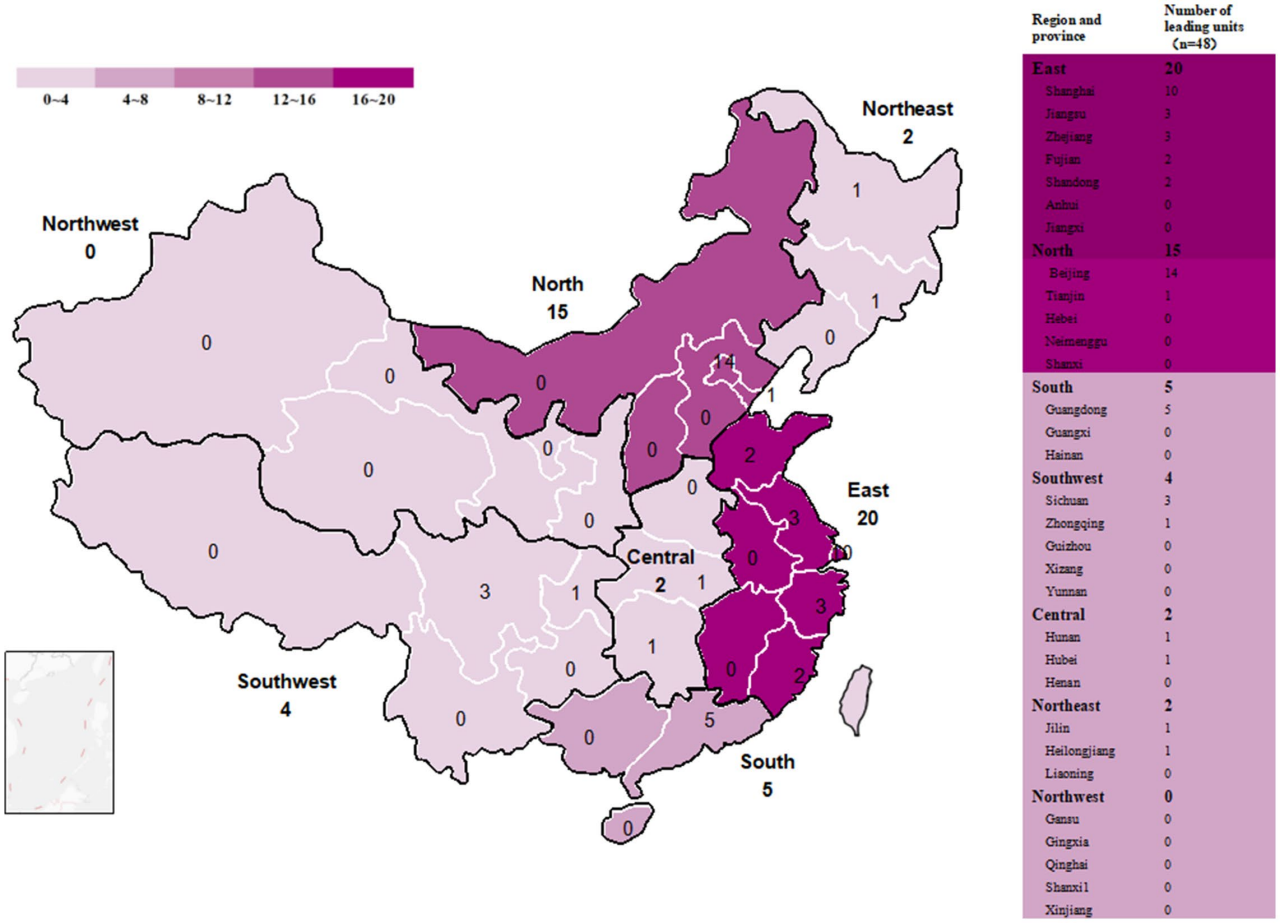
Distribution of leading unit institutions in mainland China.

We found that 48 institutions participated in pediatric clinical trials in mainland China as leading unit institutions. Among them, Beijing Children’s Hospital, Capital Medical University, had the most clinical trials with 37 trials, followed by Tongji Hospital, Tongji Medical College of HUST, with 23 trials. Two medical institutions participated as the leading unit institutions in more than 10 clinical trials: Peking University First Hospital (18 trials) and Tianjin Hematonosis Hospital (15 trials). The geographic distribution of the leading institutions is generally consistent with our previous findings (3); East China had the highest number of leading unit institutions (41.6%), followed by North China (31.2%), Southwest China (10.4%), South China (8.3%), Central China (4.2%), and Northeast China (4.2%) (Figure 10).

## 4. Discussion

### 4.1. Characteristics of innovative pediatric drug clinical trials in China

#### 4.1.1. The number of clinical trials has grown significantly, but the overall number and level of innovation remain insufficient

Restricted by the development level of the times, China did not pay sufficient attention to the importance of innovation in drug research and development in the past, and generic drugs were often the first choice of companies. In recent years, under a series of incentive policies, Chinese pharmaceutical companies producing innovative drugs and innovative drug R&D have progressed significantly. Simultaneously, the development of innovative pediatric drugs in China has also made significant progress. Since 2017, the number of innovative pediatric drug clinical trials in China has increased significantly; the number of trials conducted from 2017 to 2021 accounts for 76.3% of the total number since 2009. After a brief decline due to COVID-19 in 2020, the number returned to pre-pandemic levels in 2021.

However, further analysis of the data revealed that the level of development of innovative pediatric drugs in China is still insufficient. First, compared with the increase in the number of overall pediatric clinical trials, the number of trials specifically for innovative pediatric drugs is relatively small. By the end of 2021, domestic innovative pediatric drug clinical trials accounted for only 24.9% of all pediatric clinical trials (198/796 trials). If overseas companies were excluded, the proportion of innovative drugs developed and initiated by domestic Chinese companies dropped further to 8.9% (71/796 trials). Notably, while the average growth rate of pediatric clinical trials from 2009 to 2020 was 44.7% (3), that of innovative pediatric drugs was only 27.8%, indicating a significant lag in innovative drug R&D. At this stage, pediatric drug R&D in China is still dominated by generic drugs. Second, there is a gap between the R&D technical level of domestic and overseas companies. Domestic companies developed less than half of the 71 first-in-class trials and focused primarily on modified new drugs. The number of modified new drug clinical trials by domestic companies was much higher than that of overseas companies, which is consistent with a lower technology requirement. With the advantages of high success rate, high return, low risk, and long-life cycle, modified new drugs may be an optimal choice for domestic companies in the transition phase from generic drugs to innovative drugs.

#### 4.1.2. R&D areas are relatively concentrated

We observed that the R&D areas of domestic companies are relatively concentrated. The number of clinical trials for two major ATC classes of drugs, systemic hormones (excluding sex hormones and insulin) preparations and sensory organs, accounted for over 50% of all clinical trials by domestic companies. Conversely, overseas companies covered a wide range of R&D areas. With the exception of systemic hormone preparations and sensory organs, overseas companies had better coverage of almost all human systems. The highly concentrated nature of domestic clinical trials is bound to cause problems regarding duplicate R&D and low drug variety. For example, 25 clinical trials for growth retardation in children covered only seven drug types, and 3.5 clinical trials were conducted for each drug on average. These drugs have similar active ingredients and dosage forms, and most of the clinical trials focused on phase II/III. This redundancy will inevitably compress corporate profits, hinder the overall improvement of domestic R&D levels, and further reduce innovation in domestic pediatric drugs.

It is worth mentioning that, compared with adult anti-neoplastic drug R&D, pediatric anti-neoplastic drug R&D by both domestic and foreign companies was insufficient. Currently, anti-neoplastic drugs are a main focus in innovative drug R&D; there were 671 types of anti-neoplastic drugs registered on the platform for clinical trials, 477 of which were innovative, from 2009 to 2018 (6). In contrast, our research shows that no domestic clinical trials of innovative drugs in pediatric oncology were conducted in China during the same period (2009–2018), and only six clinical trials were conducted after 2018. However, this does not mean that the clinical need for anti-neoplastic drugs in children is lower than that in adults; even in developed countries, cancer remains the leading cause of death in children over 1 year of age (9). Statistics show that from 2018 to 2020, an average of 40,380 children and adolescents were newly diagnosed with cancer each year, and the 3-year average total incidence was 126.48 million in China (10). Compared with the adult cancer prevalence rate of 186.46/100,000 in China (11). Although the prevalence rate of cancer in children is relatively low, our study shows that pediatric anti-neoplastic drug R&D is severely lacking both at home and abroad. The relative lack of pediatric anti-neoplastic drugs may reflect the difficulty of development and market risk associated with differences in pathogenesis, tissue origin, and genetic drivers between pediatric and adult tumors.

#### 4.1.3. Subject coverage remains inadequate

A 2017 study of guardians of hospitalized children in four hospitals showed that only 4.9% of guardians had experience with their wards participating in pediatric drug clinical trials, and 6.4% had a desire to involve their wards in pediatric drug clinical trials (12). Therefore, one of the challenges facing pediatric clinical trials is the difficulty in recruiting children. According to our data, there has been some improvement in the recruitment of pediatric subjects; and the subjects in 43.9% of clinical trials were all teenagers and younger. Due to the lack and immaturity of various enzymes, children are often more prone to adverse drug reactions than adults. According to the National Adverse Drug Reaction Monitoring Annual Report, a total of 2,023,000 cases of adverse drug reactions occurred in China in 2022, of which children under 14 years of age accounted for 7.8%. This risk may be more pronounced, especially during the pre-marketing clinical study phase, when drug safety has not been verified by reliable clinical data (13). Clinical trial insurance and data monitoring have strengthened the protection of subjects and have contributed to improving trial quality. Although the DMC establishment rate for innovative drug trials has improved compared to that of non-innovative drug trials (29.3% VS. 9.9%), domestic companies have been relatively slow to implement DMCs (9.8%). This may be a result of imperfections in relevant domestic laws and regulations, insufficient DMC professional technical teams, and the lack of DMC technical guidelines. With the NMPA officially joining the International Council for Harmonisation of Technical Requirements for Pharmaceuticals for Human Use (ICH) in 2017, the concept of drug clinical trial supervision in China has begun to align with international standards. In 2020, the Center for Drug Evaluation of the NMPA released the Guidelines for Drug Clinical Trial Data Monitoring Committee (for Trial Implementation), representing the first DMC-related technical guidance document in China (14). The continuous improvement of these laws, regulations, and technical guidelines will create a sound policy environment for DMC development in China. The rate for clinical trial insurance but has of domestic companies was lower than that of overseas companies, it had improved over the last several years. The gap between domestic and foreign companies is expected to narrow in the future with further improvements to domestic laws and regulations and public awareness of rights.

#### 4.1.4. The number of pediatric clinical trial institutions is small, and the distribution of resources is uneven

In our previous study, we found that, although the number of pediatric clinical trial institutions has increased significantly in recent years (3), the absolute number of pediatric clinical trial institutions is still insufficient and uneven geographical distribution compared with adult clinical trial institutions. This problem is more prominent in pediatric clinical trials of innovative drugs, especially the unbalanced distribution of resources, with East China and North China accounting for more than 70% of the country’s leading units and no leading units in Northwest China. Within the major regions, the distribution of resources was also uneven; a large number of clinical trial resources were controlled by only a few leading large medical institutions (Figure 10). With abundant medical resources and well-constructed clinical trial facilities, large medical institutions can play a positive role in improving the quality of clinical trials; however, the excessive concentration of clinical trials may lead to problems such as lowered standards, insufficient time invested by researchers, and difficulties in recruiting subjects in hot-spot areas. Policymakers should take steps to optimize resource allocation to medical institutions in disadvantaged regions to gradually increase their output.

### 4.2. The Chinese government’s efforts in pediatric innovative drug research and development

The R&D of innovative drugs requires extremely high R&D capacity and huge capital investment, and pediatric innovative drugs often have greater risk and a longer reporting cycle. For a long time, China’s pediatric innovative drugs have had neither the support of a favorable external environment nor the motivation of self-breakthrough, and the development of the industry has been limited. In this situation, policy incentives and support seem to be the key to increasing pediatric innovative drug R&D. In fact, in recent years, pediatric innovative drug R&D have indeed received strong support from the Chinese government at all levels. Here, we briefly introduce the measures taken by the Chinese government to promote pediatric innovative drug R&D.

#### 4.2.1. Accelerated review and approval of innovative pediatric drugs to speed up the development and launch of innovative drugs

In recent years, the Chinese government has continued to implement policy measures to encourage R&D innovation and improve review efficiency. The Drug Administration Law of the People’s Republic of China, which was amended in 2019, explicitly encourages the development and innovation of pediatric drugs and prioritized their review and approval (15). The Provisions for Drug Registration, which came into effect in 2020, establish priority review channels to accelerate the listing registration process and included pediatric drugs in the priority review and approval process (4). In 2021, of the 115 registration applications included in the priority review and approval process, new types of pediatric drugs accounted for 29.6% (16). In addition, to speed up the entry of overseas innovative drugs into China, the Chinese government has developed three catalogs of new overseas drug types in urgent clinical need for three consecutive years since 2018 and has encouraged overseas enterprises to submit applications, for which it established devoted channels for review and approval (17–19). Three batches for the catalog of overseas new drugs in urgent clinical need included 81 drugs; 11 pediatric drugs (13.6%) and six pediatric innovative drugs, such as Canakinumab and Dinutuximab beta, have been successfully approved for marketing in China through this channel. These drugs have helped meet the clinical treatment needs of pediatric patients with rare and life-threatening diseases (20).

#### 4.2.2. Strengthened evaluation and technical guidance system for pediatric clinical trials

Owing to the pediatric population, companies often face technical difficulties in the process of pediatric drug development, such as formulation design, effectiveness evaluation, and clinical trial design. In recent years, the NMPA has established a system for reviewing evidence of pediatric drug development, which includes guidelines for pharmacokinetic studies in pediatric populations, extrapolation of adult medication data, and real-world data support. In 2021 alone, the NMPA released 12 special guidelines for pediatric drugs, including “Guidelines for the pharmacological development of pediatric drugs (chemical drugs) (for trial implementation) (21),” which involved real-world studies, clinical pharmacology studies, and clinical trials of improved new drugs. The aims of these guidelines were to constantly improve clinical trials and safety evaluation standards for pediatric drugs and provide important technical support and a review basis for pediatric drug development. In addition, after China joined the ICH, the NMPA accelerated the implementation of the relevant technical guidelines of the ICH and required the development of Chinese pediatric drugs to be in line with advanced international experience. Thus, pediatric drug R&D and clinical trials became more standardized (22).

## 5. Conclusion

The imperfections of early pharmaceutical policies, low investment in new drug R&D, lack of corporate innovation, and weak clinical research level made Chinese innovative drug development lag behind that of overseas companies. Although the Chinese government has continuously enhanced the power of incentive policies for innovative pediatric drugs, innovative pediatric drugs have made progress, there are still shortcomings in the quantity and level of R&D, quality of clinical trials, resource investment, and distribution of innovative drugs in China due to objective factors, such as technical difficulties and return expectations, which leave a large gap between China and the global level. Children’s health is the foundation of national health and an important guarantee for sustainable economic and social development. Guiding more resources to invest in the field of innovative pediatric drugs and improving R&D in the pharmaceutical industry are challenges faced by the Chinese government. Thus, the Chinese government should build a complete monitoring framework with more financial incentives and mandatory requirements for innovative pediatric drugs. Furthermore, China should be in line with other developed countries and work toward increasing the list of essential drugs for children, pediatric clinical trial networks, and international cooperation in pediatric research.

## Data availability statement

The original contributions presented in the study are included in the article/Supplementary material, further inquiries can be directed to the corresponding authors.

## Author contributions

W-WW, N-YM, and XJ planned and led the study and wrote the original draft of the paper. W-WW and JX undertook the literature search and applied the eligibility criteria. W-WW, X-SM, X-YM, and Y-TH retrieved the clinical information from the Chinese Clinical Trials Registry and Information Transparency Platform. W-WW, J-YZ, JX, and X-RX undertook data extraction. W-WW and N-YM designed and applied the statistical methods. All authors participated in the preparation, review, and editing process of this paper and have approved the final article.

## Funding

This study was supported by the Hospital Pharmacy Foundation of Nanjing Pharmaceutical Association–Changzhou SiYao Pharmaceuticals (2020YX024) and Jiangsu Pharmaceutical Society–Hengrui Hospital Pharmaceutical Fund research project (H202124).

## Conflict of interest

The authors declare that the research was conducted in the absence of any commercial or financial relationships that could be construed as a potential conflict of interest.

## Publisher’s note

All claims expressed in this article are solely those of the authors and do not necessarily represent those of their affiliated organizations, or those of the publisher, the editors and the reviewers. Any product that may be evaluated in this article, or claim that may be made by its manufacturer, is not guaranteed or endorsed by the publisher.

## References

[1] Zhang HC Liu ZX Wen JM. More than 200 million children are in dire need of medicines. Chinese Contemporary Medicine (2016) 23:6–7.

[2] White paper on the investigation of pediatric drug use safety (2016). Available at: https://www.ruiwen.com/gongwen/diaochabaogao/99076.html (accessed October 9, 2022).

[3] Wu WW Ji X Wang H Chen F Ding Q Zhang GD. Pediatric clinical trials in mainland China over the past decade (from 2009 to 2020). Front Med (Lausanne). (2021) 8:745676. doi: 10.3389/fmed.2021.745676, PMID: 34671625 PMC8520938

[4] National Medical Products Administration Provisions for drug registration. (2020). Available at: https://www.nmpa.gov.cn/xxgk/fgwj/bmgzh/20200330180501220.html (accessed October 9, 2022).

[5] National Medical Products Administration Reform scheme of the classification system for registration of chemical drugs. (2016). Available at: https://www.nmpa.gov.cn/yaopin/ypggtg/ypqtgg/20160309151801706.html (accessed October 9, 2022).

[6] Li N Huang H-Y Wu DW Yang ZM Wang J Wang JS. Changes in clinical trials of cancer drugs in mainland China over the decade 2009–18: a systematic review. Lancet Oncol. (2019) 20:e619–26. doi: 10.1016/S1470-2045(19)30491-7, PMID: 31674320

[7] National Health Commission of the People’s Republic of China First list of rare diseases. (2018). Available at: http://www.nhc.gov.cn/cms-search/xxgk/getManuscriptXxgk.htm?id=393a9a37f39c4b458d6e830f40a4bb99 (accessed October 9, 2022).

[8] List of Reference on Rare Diseases in China (2018). Available at: http://www.cord.org.cn/ (accessed October 9, 2022).

[9] Tang L Gao LC Ma XL Wang XL Chen XM Zou LM. Current situation and consideration of development of pediatric antitumor drugs. Chin J Clin Pharmacol. (2021) 37:1296–00. doi: 10.13699/j.cnki.1001-6821.2021.10.037

[10] Ni X Li Z Li X Zhang X Bai G Liu Y. Socioeconomic inequalities in cancer incidence and access to health services among children and adolescents in China: a cross-sectional study. Lancet. (2022) 400:1020–32. doi: 10.1016/S0140-6736(22)01541-0 36154677

[11] Rongshou Z Zhang S Zeng H Wang S Sun K Chen R. Cancer incidence and mortality in China, 2016. J Natl Cancer Center. (2022) 2:1–9. doi: 10.1016/j.jncc.2022.02.002 PMC11256658 39035212

[12] Lixia L Xiaotong L. Investigation on guardians’ cognition and experience of involvement in pediatric drug clinical trials. J Pediatr Pharm. (2021) 27:39–42. doi: 10.13407/j.cnki.jpp.1672-108X.2021.08.012

[13] Center for Drug Reevalution Annual report on national adverse drug reaction monitoring (2022). (2023) Available at: https://www.cdr-adr.org.cn/drug_1/aqjs_1/drug_aqjs_sjbg/202303/t20230324_50019.html (accessed May 3, 2023).

[14] National Medical Products Administration Guidelines for data monitoring committees for drug clinical trials (tryout). (2020). Available at: https://www.cde.org.cn/main/news/viewInfoCommon/5db2c8039ee431f074451f3f2ea42e00 (accessed October 9, 2022).

[15] People’s Republic of China of State Council Drug administration law of the People’s republic of China.(2019). Available at: http://www.gov.cn/xinwen/2019-08/26/content_5424780.htm (accessed October 9, 2022).

[16] National Medical Products Administration, Center for Drug Evaluation The pharmaceutical examination reform blossomed again and again, and the examination and approval yielded fruitful results. (2022). Available at: https://www.cde.org.cn/main/news/viewInfoCommon/e2413193d64f4541672c42e959987139 (accessed October 9, 2022).

[17] National Medical Products Administration, Center for Drug Evaluation Notice on the release of the list of the first batch of new overseas drug types in urgent clinical need. (2018). Available at: https://www.cde.org.cn/main/news/viewInfoCommon/21de8acd6c395746b041b2ad93eb5c43 (accessed October 9, 2022).

[18] National Medical Products Administration, Center for Drug Evaluation Notice on the release of the list of the second batch of new overseas drug types in urgent clinical need. (2019). Available at: https://www.cde.org.cn/main/news/viewInfoCommon/82f3bf94dc2c38d1a24d851f0e44914b (accessed October 9, 2022).

[19] National Medical Products Administration, Center for Drug Evaluation Notice on the release of the list of the third batch of new overseas drug types in urgent clinical need. (2020). Available at: https://www.cde.org.cn/main/news/viewInfoCommon/08818b168ccc85db9a42a0f6623b5688 (accessed October 9, 2022).

[20] National Medical Products Administration, Center for Drug Evaluation Leading Children’s drug use to spring—a record of the work of the NMPA to improve the availability and safety of children’s drug use. (2022). Available at: https://www.cde.org.cn/main/news/viewInfoCommon/bd6e5fa36b1ccfc05bfd47c31c73bfd8 (accessed October 9, 2022).

[21] National Medical Products Administration, Center for Drug Evaluation Combined fist’ to help solve the problem of drug use in children. (2022). Available at: https://www.cde.org.cn/main/news/viewInfoCommon/701873e3d25e34d2a460b03a12f90899 (accessed October 9, 2022).

[22] National Medical Products Administration, Center for Drug Evaluation NMPA has taken multiple measures to encourage innovation, and the review and approval of children’s drugs continues to grow. (2022). Available at: https://www.cde.org.cn/main/news/viewInfoCommon/62ac636f7e25fc3913e513d21066d684 (accessed October 9, 2022).

