# Supplementary material for: Trends in innovative pediatric drug development in China based on clinical trial registration data
Source: Front Med (Lausanne). 2023 Jul 6;10:1187547. doi: 10.3389/fmed.2023.1187547 (PMC10359815; doi:10.3389/fmed.2023.1187547)
Supplement: Supplementary file 1 [file Data_Sheet_1.PDF]

Supplementary document table 1 classification of innovative drugs

Classification	Chemical drugs				Therapeutic biological products	
	New registration types	Definition/scope	Old registration types	Definition/scope	New registration types	Definition/scope
**First-in-class drugs**	Class 1	Innovative drugs, with new defined structures, pharmacology and clinical value, which have never been marketed within or outside China.	Class 1.1	Drug substances and their preparations produced by synthetic or semi-synthetic methods and have never been marketed within or outside China.	Class 1	Therapeutic biological products that have never been marketed within or outside China.
			Class 1.2	New effective monomers and their formulations derived from natural substances or extracted by fermentation that have never been marketed within or outside China.		
**Modified new drugs**	Class 2.1	Drug substances and preparations with obvious clinical advantages: optical isomers, with known active ingredients, produced by splitting or synthesis methods; esterification or salt formation of known active ingredients	Class 1.3	Optical isomers and preparations of known drugs produced by splitting or synthesis methods that have never been marketed within or outside China.	Class 2.1	Biological products, with obvious clinical advantages, whose dosage forms and routes of administration are optimized based on the marketed products.
			Class 4	Drug substances and their preparations whose acid radical, base or metal elements of salt drugs marketed are changed but pharmacology remain.		

		(including salts containing hydrogen bonds or coordination bonds); modification of acid radical, base or metal elements of known salt active ingredients; or formation of other non-covalent bond derivatives (such as complex, chelate or inclusion compound).				
	Class 2.2	Preparations of known active ingredients with new dosage form (including new drug delivery system), formulation or route of administration; with obvious clinical advantages.	Class 2	Preparations with new routes of administration that have never been marketed within or outside China.	Class 2.2	Biological products for new indications which have not been approved either domestically or internationally and new targeted population.
			Class 5	Preparations with new dosage forms and original routes of administration that have been marketed within or outside China.		
	Class 2.3	New compound formulations of known active ingredients with obvious clinical	Class 1.4	Less-component drugs obtained from multi-component drugs on the market that have never been marketed within or outside China.	Class 2.3	New compound products combined by biological products with similar ones on the market.

		advantages.	Class 1.5	New compound preparations that have never been marketed within or outside China.		
	Class 2.4	Preparations with known active ingredients for new indications.	Class 1.6	Preparations have been marketed within or outside China for new indications which have not been approved either domestically or internationally.	Class 2.4	Biological products with significant technical improvement based on marketed products, e.g., Recombinant technology instead of biological tissue extraction technology; biological products with obvious clinical advantages through changing amino acid sites, expression systems or host cells of marketed products.
**Overseas innovative drugs**	Class 5.1	Originator drugs (including drug substances and their preparations) that had been marketed outside of China and now apply for being marketed in China.			Class 3.1	Biological products produced and marketed overseas that have not been marketed domestically and now apply for being marketed in China.

Supplementary document table 2 indications and quantitative distribution of 198 clinical trials of pediatric innovative drugs in China

Indication	No. of trials	Ratio	Indication	No. of trials	Ratio	Indication	No. of trials	Ratio
Growth retardation in children or microsomia	28	14.1%	Graft rejection reaction	2	1.0%	X-linked hypophosphatemic rickets/osteochondrosis（XLH）	1	0.5%
Hemophilia A	16	8.1%	Chronic hepatitis B	2	1.0%	Lennox-Gastaut syndrome	1	0.5%
Epilepsy	14	7.1%	Multiple sclerosis	2	1.0%	Acute otitis externa	1	0.5%
Asthma	12	6.1%	Acute hyperuricemia in patients with high tumor	2	1.0%	Pneumonia	1	0.5%
Atopic dermatitis	9	4.5%	Acute lymphocytic leukemia	2	1.0%	Alopecia areata	1	0.5%
Juvenile idiopathic arthritis	8	4.0%	Mucopolysaccharidosis	2	1.0%	Dermatitis eczema	1	0.5%
Myopia	6	3.0%	Dravet syndrome	2	1.0%	Acne vulgaris	1	0.5%
Tumor	6	3.0%	SMA	2	1.0%	Gaucher disease	1	0.5%
Syncytial virus infection	5	2.5%	Pediatric sedation	2	1.0%	Pompe disease	1	0.5%
Hemophilia（not specified）	5	2.5%	Immune thrombocytopenic purpura（ITP）	2	1.0%	Postoperation of portoenterostomy in patients with biliary atresia	1	0.5%
Pulmonary artery hypertension	5	2.5%	Nevus flammeus	2	1.0%	Heart failure in children	1	0.5%
Diabetes	4	2.0%	Turner syndrome	2	1.0%	Atypical hemolytic uremic syndrome（aHUS）	1	0.5%
Allergic rhinitis	4	2.0%	Tourette syndrome	2	1.0%	Primary immunodeficiency（PID）	1	0.5%
Influenza	4	2.0%	Anesthesia	1	0.5%	Prader-Willi syndrome	1	0.5%
Central precocious puberty	4	2.0%	Infectious diarrhea	1	0.5%	Thrombopenia	1	0.5%
Duchenne muscular dystrophy	3	1.5%	Urticaria	1	0.5%	Neonatal respiratory distress	1	0.5%
Auxiliary diagnosis	3	1.5%	Crohn's disease	1	0.5%	Bacterial infectious disease	1	0.5%
Infantile hemangioma	2	1.0%	Respiratory tract infections in children	1	0.5%			
Thalassemia	2	1.0%	Systemic lupus erythematosus	1	0.5%			
ADHD	2	1.0%	Achondroplasia	1	0.5%			
Schizophrenia or bipolar disorder	2	1.0%	Venous thromboembolic events (VTE)	1	0.5%			
Hemophilia B	2	1.0%	Iron-deficiency anemia	1	0.5%			
						Total	198	100%
